# Physiologische, Pathologische und Medicinisch-practische Untersuchungen uber die Spinal Irritation

**Published:** 1844-07-01

**Authors:** 


### PHYSIOLOGISCHE, PATHOLOGISCHE UND MEDICINISCH-PRACTISCHE UNTERSUCHUNGEN UBER DIE SPINAL IRRITATION. Von Dr. *Stilling*. Leipzig, 1840.

Physiological, Pathological, and Practical Investigations regarding Spinal Irritation.

### ABHANDLUNG UBER SPINAL IRRITATION. Von Dr. *Ludwig Türck*. Vienna, 1843.

Treatise on Spinal Irritation.

WE find that the latter of the above works contains in itself a very neat and succinct analysis of the former, which was sent into the world in 1840 by Dr. Stilling, who is now celebrated for his writings on nervous disorders in general.

It has been recently observed, that in certain of the neuroses, more especially in hysterical states, a greater or less extent of surface of the parts along the vertebral column becomes painful on pressure and on being touched with a hot sponge; it was also observed that these states were frequently removed, or at least considerably relieved, by abstraction of blood, counter-irritation, &c. along the painful vertebræ; this very naturally led medical men to seek the cause of those nervous symptoms in a state of irritation of the spinal marrow; the affection itself receiving the name of *spinal irritation*. Among others, Dr. Türck was led to institute a series of observations on this subject, the results of which are given in the volume now before us. The cases where the spinal irritation is to be considered merely as a reflexion of other diseases existing in the organism he treats of under the head of "*Symptomatic Spinal Irritation*;" whilst those cases wherein this affection still continues after the extinction of the primary disease, or where its dependence on some disease existing elsewhere cannot be demonstrated, he treats of under the title of "*Idiopathic Spinal Irritation.*" The exposition of the facts observed by him is followed by an investigation into their nature. We shall here present to our readers a concentrated analysis of this volume. It was already observed by several that by pressure, even by mere rubbing of the sensitive parts along the spinal column, some symptoms present in spinal-irritation were aggravated; this has been observed with respect to pains in the head and in the eyes, in the scrobiculus cordis and chest, with respect also to cough, a sensation of cold, stiffness of the arms, and pain in the neck, also in some cases, which belong to the division under idiopathic spinal irritation. The aggravation or recalling of symptoms by vertebral pressure, the author designates in his treatise by the term "reflexion," and the parts along the vertebral column, where the pressure produced this effect, he calls reflex-parts.

### First Section.

### 1. *Symptomatic Spinal Irritation.*

A. *Diseases of the Digestive Apparatus.*—In some cases of caries of the back and incisor teeth and in inflammation of the gum, the pains were reflected through the second down to the third cervical vertebra and the soft parts adjacent to the affected side. In gangrene of the mouth connected with violent inflammation in consequence of swallowing sulphuric acid, the painful constriction behind the larynx was reflected through the fourth to the sixth cervical vertebra. In *gastricism, febris gastrica*, the frontal and supra-orbital pain was reflected through the superior fourth down to the fifth cervical vertebræ, attended on one occasion with a peculiar disagreeable sensation in the stomach and a disposition to vomit. In another case, the disagreeable sensation in the stomach was reflected through the last cervical and first dorsal vertebræ. Acute, pungent pains of a higher degree in the epigastrium were reflected with great sensibility to external pressure through the last third or fourth dorsal vertebra; where the pains were less, this did not occur. On one occasion temporary numbness of the left arm was present. In a case of sporadic cholera in a hysterical patient, violent, colicky, constringing pains, increased on pressure, were felt all around the abdomen, which were reflected with severe pungency in the last ribs of the left side through the very sensitive lumbar vertebræ; the respiration was frequent, there was frequent, dry cough and dyspnœa at the same time, the last with reflexion through the inferior half of the dorsal vertebræ; pulse not very frequent, urine pale. In typhus, vertigo, tinnitus aurium and head-ache were reflected through the superior half of the cervical vertebræ. The sensation of dryness of the tongue and thirst were increased once or twice by pressure on from the fourth to the sixth cervical vertebræ; the pasty, bad taste in the case of loaded tongue in the first stage of the disease was on one occasion almost entirely removed thereby. Delirium, feeling of weakness, tremors and singultus were without any reflexion. In inflammation of the peritoneal covering of the liver, in increased sensibility, and tenseness in the region of the left lobe, with icteric and gastric phenomena, the acute pain in the right humeral and clavicular region and numbness of one extremity was reflected twice through the fourth and fifth dorsal vertebræ, once through the last cervical vertebra; in the same case also the pain in the hepatic region was reflected through the first dorsal vertebra.

B. *Diseases of the Organs of Respiration and Circulation.*—Dryness, pungent pain in the larynx, as also hoarseness, which accompanied the other symptoms in tuberculosis, were reflected through the fourth or fifth last cervical vertebræ. Tickling at the bifurcation of the bronchi, and teazing cough, such as attends catarrh and tuberculated lungs, were reflected through from the third to the seventh cervical vertebræ. The head-symptoms observed most frequently in pneumonia were: acute pain in the fore-head and temples, heaviness in the frontal region, tinnitus aurium, with reflexion through the superior cervical vertebræ, at most as far as the fifth; delirium with vertebral reflexion. If several of these were present at the same time, and were reflected by pressure on one and the same place, then, when great debility was present, with dryness and heat of tongue and skin, they formed the perfect idea of the so-called *nervous state.* In one case there was, with acute pain in the frontal region, intolerance of light, a sensation of pressure and heat in the eyes, the latter symptoms attended with reflexion through the last cervical vertebræ; in another case, the sensation of itching in the thoracic region on the side corresponding to the seat of the pneumonia, with reflexion through the last cervical vertebra, on two occasions vertigo with reflexion through the highest part of the vertebral column beneath the occiput; on one occasion, a rising of heat in the cheek with reflexion through the first dorsal vertebra. Pungent pain at the sternal extremities of from the second to the sixth costal cartilage combined with the sensation of pressure at the same places and at the sternum, was frequently increased on pinching the skin of those parts, and felt as if it were deeply seated; further it came along the cartilaginous margins of the false ribs; lastly in an extent of from one to two inches in length at the free extremities of the last ribs, once at the inferior margin of the seventh and eighth ribs along their entire course, the intercostal spaces being free from pain, once only at the curvature of the sixth and seventh ribs; it was generally increased by pressure on the painful parts, and was reflected through the vertebræ corresponding to the ribs affected over these and through the adjacent soft parts. The sensation of pressure on the sternum, commonly on one or both margins of the same, and on the sternal ends of from the second to the ninth costal cartilage, which was frequently combined with acute pains in these parts, was, as well as the latter, reflected through the corresponding dorsal vertebra. In two cases dyspnœa, where it was the only subjective thoracic symptom, was reflected through the inferior cervical and superior dorsal vertebræ, in one of these cases the reflexibility extended from here downwards as far the inferior dorsal vertebræ; it occurred in general accompanied by pain and pressure on the chest, and was reflected along with them; frequently the reflexibility for the dyspnœa was more extended than for the other thoracic symptoms; it was, however, almost invariably greatest at the last cervical and superior dorsal vertebræ. The author observed, both in pneumonia as well as in other diseases connected with dyspnœa, that by pressing on the reflex-parts of the dyspnœa the patients were less able to take a deep inspiration. On one occasion, in a very sensitive young man, cough was reflected through the inferior cervical and superior dorsal vertebræ and indeed with such violence, that he gave over the examination on the other vertebræ. Not rarely was the epigastrium very sensitive on pressure and the seat of very severe, acute pains in two cases, which were increased during inspiration, and often by pinching the skin, or gently rubbing it in the above-mentioned regions, and were felt as if they were very deep-seated. They extended to the adjoining costal cartilages as far as the hypochondria, occurred most frequently in the case of pneumonia of the right side, and were reflected through the upper dorsal vertebræ, at most as far as the sixth or eighth, once however through the inferior dorsal vertebræ, being generally accompanied with dyspnœa. Pungent pain in the left lobe of the liver was frequently combined with pains in the epigastrium; this pain did not admit of pressure; nor was it ever reflected. In two cases the painfulness on pressure extended over the principal part of the abdomen. On one occasion there were tolerably intense pains along the last five ribs, and in the adjacent muscular parts, and still deeper in the lumbar region, from whence they extended along the crest of the ilium as far the inguinal region; they were at the same time increased by pressure on the painful parts, and reflected through the lumbar vertebræ corresponding to their seat, and, as well as the increased sensibility of the abdomen, were present only on the side corresponding to the seat of the pneumonia. Numbness of the extremities was no rare occurrence; it affected either all the four extremities, or only those of one side, twice only the right arm. In this case, as well as where merely the upper or lower extremity of the right side participated, inflammation of the right lung with pain in the epigastrium and left lobe of the liver was constantly present along with the bilious phenomena already mentioned; in two such cases there was associated pain in the right clavicular and lateral cervical region, once also in the trachea and larynx, with reflexion through the last cervical and superior dorsal vertebræ. *In tuberculated lungs* the head and thoracic symptoms were about the same as in pneumonia. The sensations of pain, dryness, constriction in the larynx, as well as the hoarseness, were reflected through the last fourth or fifth cervical vertebræ. The abdominal symptoms observed in pneumonia were not observed here by the author. The author continues to consider the various reflexions accompanying hæmoptoe, pleuritis, endocarditis, &c. He then proceeds to the consideration of *Idiopathic Spinal Irritation*, and the first example he gives of that is *Febris Intermittens*. The instances of reflexion in the stages of this disease being neither numerous nor interesting, we shall pass on to the next affection noticed by our author, viz. hysteria; in this affection the prominent symptoms, as pain in the frontal and supra-orbital regions, pains in the back of the head and vertex, vertigo, sensation of heat ascending from the back of the head over the vertex as far as the forehead and cheeks, dimness of vision, &c. were in the generality of the cases usually combined the one with the other, and were at the same time reflected, and chiefly through the superior cervical vertebræ; frequently the capability of reflexion extended itself from thence over the chief part of the cervical, and often of the dorsal vertebræ. Sometimes delirium set in without reflexion. The feeling of constriction in the neck was reflected by from the fourth to the sixth cervical vertebræ. The dyspnœa with other thoracic symptoms was reflected for the most part through a greater or less number of the dorsal vertebræ.

Our author now proceeds to the SECOND SECTION, *in which he attempts to account for the phenomena of Spinal Irritation.* He states, of spinal irritation in general, that it arises for the most part from this circumstance, viz. that the spinal marrow becomes abnormally affected either through the nerves passing through it from the organ originally diseased, which may be called excitory nerves, or through the blood circulating in its capillary vessels.

This affection, or irritation, as we may call it, extends farther from the insertion-places of the excitory nerves in the spinal marrow, and produces, by touching the places where other nerves arise, various nervous symptoms whose apparent seat is in the parts supplied by these, whilst they solely result from an affection of the central extremities of the nerves, and are accordingly excentric phenomena. By the irritation extending to the spinal origins of the sensitive nerves of the posterior parts of the neck and trunk, *i. e.* of the dorsal nerves, it produces spinal sensibility. The chief arguments adduced in support of this view by Stilling, are: 1. Pain on pressure manifests itself in vertebræ as immovable, and where, therefore, the pain cannot be the consequence of a mechanical effect produced on the spinal marrow by locomotion of the vertebræ. 2. The spinal pain is often occasioned, not by pressure on the vertebræ, but on the soft parts lying near it. 3. It is frequently occasioned by gently touching the skin in the vicinity of the vertebræ. The reflexion of the symptoms is accounted for, in fine, by the propagation of the excitement occasioned by pressure in the sensitive dorsal nerves, through the medium of the spinal marrow as far as the origins of those nerves, which are the seat of the reflected symptoms. (The latter, the author calls reflex symptoms, and the nerves, which are their seat, reflex nerves). Between the reflex symptoms an essential distinction is to be made; some being excentric phenomena, a mere effect of spinal irritation, as, for instance, numbness of the extremities in pneumonia; whilst the others are no more than mere sensations, which have their seat in the organ primarily diseased (as for instance, pains in the ovaries or ovaritis); as the nerves of these organs produce spinal irritation in the parts where they are connected with the spinal system, it happens that the latter symptoms are reflected. The latter, therefore, are called *improper*, the former, on the contrary, *proper reflex symptoms.*

*The seat of Spinal Irritation corresponds ordinarily with the painful or reflecting parts in the Spine;* the reason of which is this, that the posterior branches of the spinal nerves, which give off the sensitive dorsal nerves, have generally a very short course, and accordingly their branches as well as their peripheric ramifications, both of which may be affected by dorsal pressure, are placed in the proximity of their origin from the spinal marrow.

According to the view now given of Spinal Irritation, the spinal marrow presents the middle point of the affection. On the contrary, the phenomena of spinal irritation may be understood in many cases, even if we leave the spinal marrow altogether out of consideration. It is known that the spinal nerves are formed of an anterior motor, and a posterior sensitive root, furnished with a ganglion; the common trunk of every spinal nerve, hence arising, contains one or more fibres of the sympathetic nerve which lies near it, which, enclosed with it in a common sheath, run along to the roots, and so come in contact with the ganglia of the sensitive roots. In those cases of spinal irritation where the fibres of the sympathetic nerve are excitor nerves, one may represent to himself, that these fibres, as they enter the spinal ganglia, create an irritation there, which becomes transferred through the medium of the grey ganglionic substance to the fibres of the sensitive nerves placed in juxta-position in these ganglia, whereby the occurrence of the reflex symptoms, the spinal sensibility and the reflexion are accounted for, because both the fibres of the reflex, as also those of the sensitive dorsal nerves, pass through the ganglia already mentioned.

The presence of sympathetic fibres in the spinal marrow, as demonstrated by Remak, does not invalidate this explanation; for—1, it is possible that only a part of the sympathetic fibres goes to the spinal marrow, and another terminates in the spinal ganglia; and, 2, if all the sympathetic fibres merely passed the spinal ganglia, a transference might be conceivable through the medium of the grey substance of these ganglia to the fibres of the sensitive spinal nerve.

In those cases, on the contrary, where the sensitive spinal nerves are excitors, it is extremely probable that the spinal marrow is the seat of the irritation, and this may be inferred, almost with certainty, from the presence of certain motory symptoms, a circumstance which makes one very much disposed to suspect an affection of the spinal cord in all cases of spinal irritation, but, which, however, cannot serve to prove this assumption, as it must now be deemed possible, that even in one and the same patient certain phenomena depend on irritation of the spinal cord, whilst others owe their origin to a mere affection of the spinal ganglia.

*Trigeminus Symptoms.*—From circumstances mentioned by the author, in his Sketches of Disease, he infers that various painful sensations in the frontal and temporal regions, and in the eyes, in cases of gastric fever, ophthalmic inflammation, and inflammation of other organs attended with fever, hysteria, &c. are reflected through the upper cervical vertebræ, more especially from the first to the third. Considering the moveability that exists between the first and second cervical vertebræ one would feel tempted to ascribe this to a locomotion of the medulla oblongata occasioned by pressure, or of the parts of the brain thereby affected; it militates, however, against this view, that in many cases reflexion occurred, not through the uppermost, but through the third and fourth vertebræ, and it frequently followed on light pressure or on pinching the skin; accordingly, in the cases instanced, spinal irritation was present in the region of the superior cervical vertebræ. Stilling maintains the above-mentioned symptoms to be excentric phenomena, dependent on irritation of the *n. trigeminus*, the sensitive nerve of the head, whose roots in the spinal marrow reached as far as the region of the second cervical vertebræ. He supports these assertions by Magendie's experiments. He saw division of the *n. trigeminus* on one side in dogs followed by anæsthesia of the correponding half of the face; the same thing happened, according to him, after dividing the spinal marrow on one side above the second cervical vertebræ; this, however, was not the case, when the division was carried deeper down between the second and third vertebræ. These experiments, and the conclusions drawn by Stilling, accord very well with the pathological facts observed by the author; for, if certain head-symptoms depend on irritation of the roots of the trigeminus reaching into the region of the second cervical verterbræ in the spinal marrow, the spinal sensibility, and the capability of reflexion in this region are accounted for in a very simple way. Notwithstanding, Stilling's view of the matter is not to be considered as proved; for, in the first place, admitting the correctness of Magendie's experiments, these were made only on dogs; in the second place, the roots of the sensitive portion of the *n. trigeminus*, though they have been traced as far as the *corpus restiforme* of the medulla oblongata, have not been traced any farther down; in the third place, the connexion of the head-symptoms above-mentioned with spinal irritation in the superior cervical vertebræ may be directly accounted for without any affection of the origins of the trigeminus in the same way as the author already accounted for the phenomena of spinal irritation without the intervention of the spinal marrow at all, by substituting the *Gasserian ganglion* for the spinal ganglia. For if it be admitted that the spinal irritation in the upper cervical ganglion is not occasioned by any irritation of the spinal marrow, but is an affection of the uppermost cervical ganglion of the n. sympatheticus, the occurrence of the head-symptoms is accounted for by the extension of the irritation from the above-mentioned ganglion to the *Gasserian ganglion* through the medium of the connecting fibres existing between both, but the spinal sensibility and capability of reflexion in the upper cervical vertebræ is then a consequence of the extension of the irritation from the uppermost sympathetic ganglion to the posterior sensible roots of the four superior spinal nerves, to which it is known there are sent connecting twigs.

The Reflexion of the pain in carious teeth, and in *neuralgia alveolaris*, through the superior cervical vertebræ, may be easily understood from the preceding, the dental nerves being branches of the trigeminus.

The Reflexion of pain as well as the sensation of heat on the vertex and occiput in hysterical patients, through the superior cervical vertebræ, is accounted for by the circumstance, that the skin of the above-mentioned regions is supplied in a great measure by the second spinal nerves, and partly, too, by the *n. trigeminus*.

In the same manner the other reflex symptoms are accounted for anatomically, as they occur in different parts of the body. We have given an account of these investigations, to put our readers au niveau of the notions of the day. For our own parts, we do not hesitate to own ourselves incredulous respecting them.

